# Foreign Body Aspiration Presenting with Asthma-Like Symptoms

**DOI:** 10.1155/2013/317104

**Published:** 2013-12-28

**Authors:** Jennifer C. Kam, Vikram Doraiswamy, Javier F. Dieguez, Joan Dabu, Matthew Cholankeril, Mayur Govind, Richard Miller, Marc Adelman

**Affiliations:** ^1^Division of Renal Diseases and Hypertension, The George Washington University School of Medicine, Washington, DC 20037, USA; ^2^Department of Internal Medicine, Saint Michael's Medical Center, Seton Hall University School of Health and Medical Sciences, Newark, NJ 07102, USA; ^3^Department of Pulmonary and Critical Care Medicine, Saint Michael's Medical Center, Seton Hall University School of Health and Medical Sciences, Newark, NJ 07102, USA

## Abstract

Aspiration of a foreign body into the tracheobronchial tree is rare in adults. In the majority of these cases there is an underlying condition such as mental retardation, depressed mental status, impairment in the swallowing reflex, neurological impairment, alcohol or sedative abuse, or complications from dental manipulations that contributed to the aspiration. These patients are commonly misdiagnosed with asthma and typically do not respond to mainstay anti-inflammatory and/or bronchodilator therapy. We describe the case of a patient with a foreign body aspiration in the upper trachea not recognized by radiographic studies that presented with asthma-type symptoms.

## 1. Introduction

Aspiration describes an event where the intake of solid or liquid materials becomes inadvertently retained in the airways and the lungs [1]. Aspiration of foreign bodies into the airways is a rare occurrence in adults but an important consideration in certain clinical presentations. The most common presenting symptoms of a foreign body in the airways include chronic cough, dyspnea, and hemoptysis that are often misdiagnosed as obstructive airway disease. Although a history of choking episodes may increase suspicion for aspiration into the airways, it may not always be observed in all patients. The swallowing reflex prevents aspiration in normal adults but the absence of this mechanism predisposes patients to consume foreign bodies into the airways [2, 3]. Risk factors for aspiration include neurological dysfunction such as stroke, encephalopathy, alcoholism, seizures, Parkinson's disease, sedatives, and mental retardation; dental procedure complications, facial trauma, intubation, and abnormalities of the pharynx and esophagus [1, 3–5]. These patients are commonly misdiagnosed with obstructive airway disease, mainly asthma, and do not respond to anti-inflammatory and/or bronchodilator therapy [6]. We describe the case of a patient with a foreign body aspiration in the upper trachea misdiagnosed as obstructive airway disease.

## 2. Case Presentation

A 34-year-old Hispanic male with a past medical history significant for asthma and bronchitis presented to the emergency room complaining of worsening dyspnea and wheezing for 3 days. He reported that his shortness of breath was present at rest and worsened with even minimal exertion. His dyspnea was associated with a productive cough with greenish-coloured sputum. He denied any fever, chills, headaches, hemoptysis, nausea, vomiting, recent sick contacts, significant travel history, or exposure to allergens.

The patient was diagnosed with asthma 5 years prior to admission and had been recently diagnosed with chronic bronchitis. He had repeated hospitalizations for exacerbations of his asthma, with his most recent being one month prior to admission. His medications on admission included Prednisone 5 mg daily, Singulair 10 mg daily, Advair 250 mcg/50 mcg inhaled daily, and an Albuterol inhaler, which he used multiple times during the day. He denied any history of smoking or the use of illicit drugs but admitted to drinking alcohol socially.

On examination, his temperature was 98.2°F, blood pressure was 126/79 mmHg, heart rate was tachycardic at 120 beats per minute, respiratory rate was 16 breaths per minute, and oxygen saturation was 98% on room air. Air entry into the lungs was decreased bilaterally with diffuse expiratory wheezes noted. No rhonchi, rales, egophony, or dullness to percussion was appreciated. He did not have edema, clubbing, or cyanosis in his extremities. His neurological exam was grossly intact. His labs were within normal limits with the exception of an elevated white blood cell count of 13,400 cells/mL with no left shift. He was admitted to the medical intensive care unit for observation where his condition failed to improve with bronchodilators or high doses of corticosteroids.

**Figure 1:**
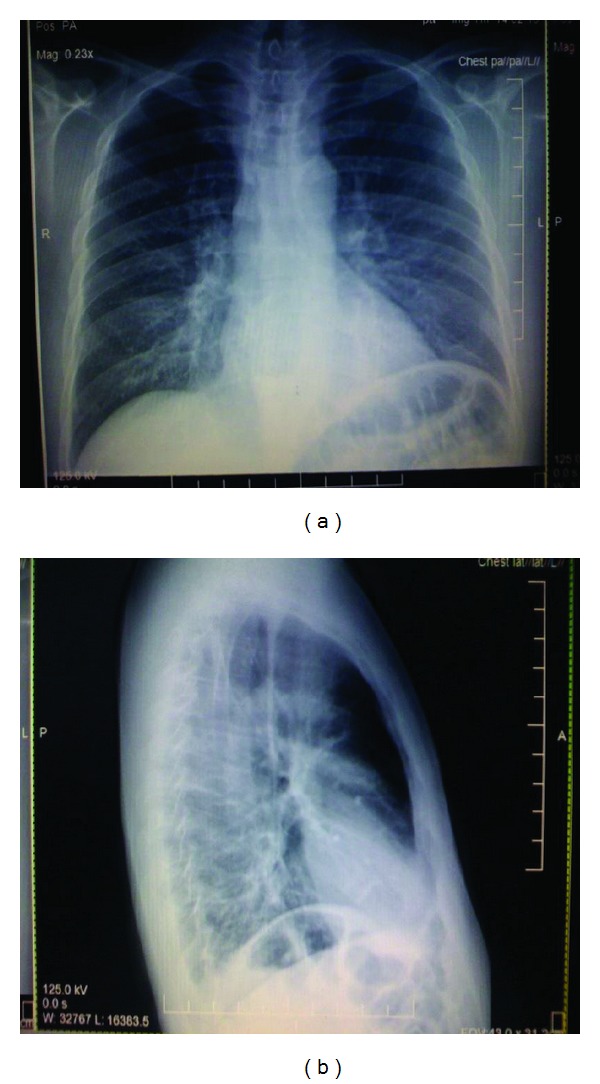
Posterioranterior and lateral chest radiographs showing dilation of the esophagus.

**Figure 2:**
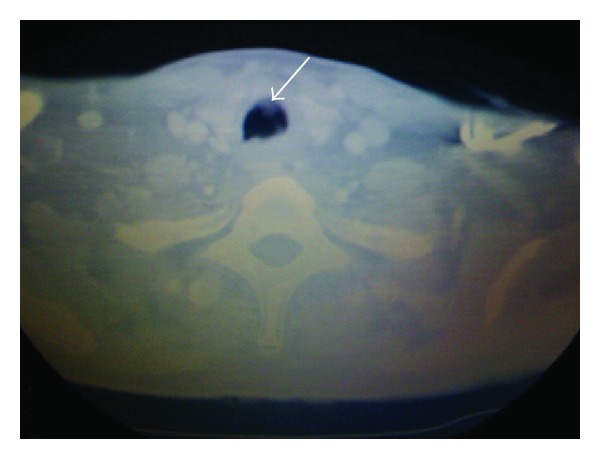
CT scan of the neck showing a haziness in the trachea representing the foreign body.

Radiography of the chest showed marked dilation of the esophagus (Figure 1). Further investigation included a CT scan of the neck revealing a pouch-like structure in the posterior esophageal wall consistent with a Zenker's diverticulum (Figure 2). Barium esophogram revealed flow of barium contrast into the lungs. The patient subsequently developed a low-grade fever and blood cultures were positive for a yeast infection. The patient underwent a Zenker's diverticulectomy and myomectomy, but the patient's condition still did not improve.

**Figure 3:**
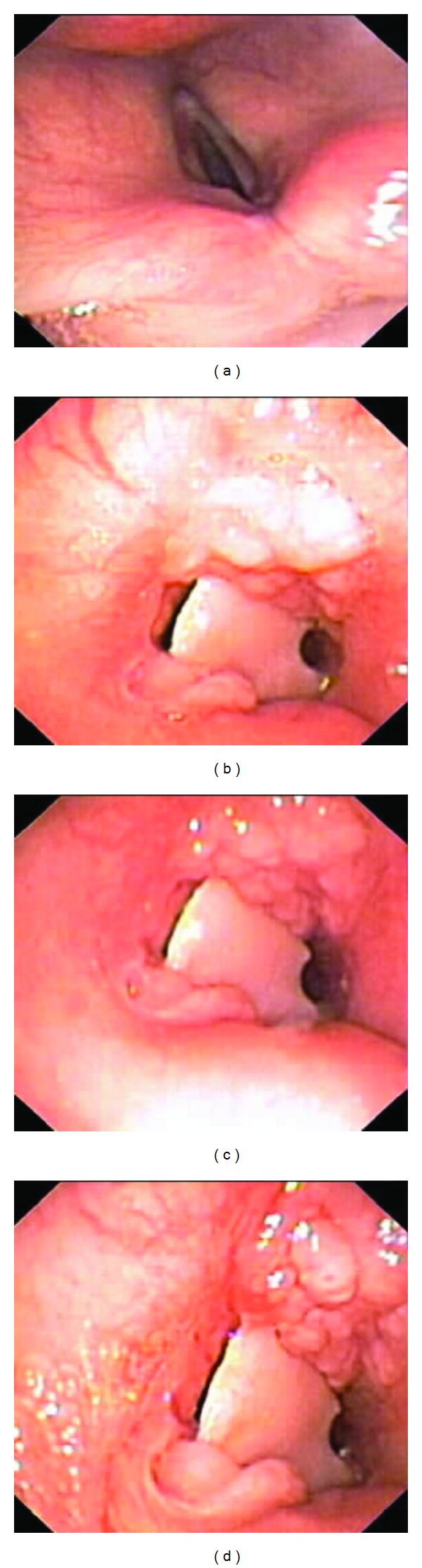
Bronchoscopic images showing vocal cords (a) and different views of the foreign body.

A repeat barium esophogram showed a tracheoesophageal fistula, as a suspected complication of the Zenker's diverticulectomy. A bronchoscopy was then performed revealing a foreign body several centimeters below the vocal cords causing approximately 90% tracheal stenosis (Figure 3). Surgical consultation was called to evaluate the obstruction. A tracheal resection was performed and a large foreign body was removed revealing the foreign body to be a complete partial denture.

On further investigation, the patient disclosed that 6 years prior, at a family reunion, he became intoxicated with alcohol and was involved in an altercation which caused him to lose a partial denture which was never recovered. Several months later he began developing signs and symptoms of asthma and was given the diagnosis of asthma. The remaining hospital course was unremarkable and the patient was subsequently discharged without further complications.

## 3. Discussion

Foreign body aspiration refers to the inhalation of solid and liquid material into the airways. The foreign body may be lodged into the main bronchi and its branches and may even reach the lung. The right main bronchial is frequently implicated because of its more vertical path. The upper lobes and superior segments of the lower lobes are commonly affected when patients are in the recumbent position [1]. Since it is a rare occurrence in adults, aspiration is commonly overlooked.

The swallowing and cough reflexes are important respiratory defense mechanisms which protect patients from aspiration [2, 3, 7]. When these mechanisms are bypassed or suppressed, it predisposes patients to aspirate foreign bodies [3]. Risk factors include alcoholism, general anesthesia, loss of consciousness, intubation, neuromuscular disorders, and structural abnormalities of the pharynx and esophagus such as tracheoesophageal fistula, Zenker's diverticulum, or achalasia [1, 3–5]. The most common aspirated foreign bodies are typically food and broken fragments of teeth [1–4].

Initial presentations of aspiration include choking sensations with an intermittent dry cough. Although choking may raise early suspicion of aspiration, it may not always be apparent with all patients [2]. Inspiratory and expiratory wheezing, hemoptysis, and dyspnea may also be observed in these patients [1–3, 8]. Chronic complications occur mainly because of a delay in correctly diagnosing foreign body aspiration, resulting in inappropriate management [1]. In many instances, the aspirated foreign body is unrecognized. Many cases with tracheobronchial aspiration are misdiagnosed as asthma or bronchiolitis as well as emphysema or atelectasis [8]. Complications include recurrent pneumonias, bronchiectasis, bronchial strictures, hemoptysis, and development of inflammatory polyps at the site of impaction [1, 3, 4, 6]. Radiographic findings in these patients are typically nonspecific but may reveal areas of increased opacities, post-obstructive infiltrate or chronic volume loss, and atelectasis [8, 9]. CT scanning of the chest may also show an intrabronchial or intraparenchymal mass [9].

The failure of asthma to respond to bronchodilators and corticosteroids should raise clinical suspicion of an incorrect diagnosis. Pulmonary function studies including a flow loop, X-ray, and CT scan of the neck may indicate alternate diagnoses. In this case, the finding of the Zenker diverticulum was misleading. The partial denture was radiolucent and even in retrospect could not be visualized radiographically. Endotracheal tube placement during intubation and surgery did not reach the site of the foreign body described and was therefore unrevealing on both occasions.

Definitive diagnosis of an aspirated foreign body can be confirmed by visualization with indirect laryngoscopy or bronchoscopy, as in our case [4, 5]. Bronchoscopic retrieval with grasping forceps can be performed with either fiberoptic or rigid bronchoscopy [2, 4] but was unsuccessful in our case due to the chronicity and size of the foreign body.

## 4. Conclusion

A foreign body aspirated within the tracheobronchial tree is a rare occurrence in adults and is often misdiagnosed as an obstructive airway disease. We present a case of aspiration of a partial denture, which caused tracheal stenosis leading to the improper diagnosis of asthma. In addition, the recognition of the denture material, which is radiolucent, could not be visualized by noninvasive means. This case shows the importance of further investigation in patients with obstructive airway disease who are unresponsive to routine therapies. The inclusion of foreign body aspiration in the differential for such patients allows for early recognition and appropriate management, thereby decreasing the incidence of costly and unnecessary complications.
